# Revealing gendered patterns and teacher competency status in online multicultural teaching competency

**DOI:** 10.12688/f1000research.177259.1

**Published:** 2026-02-11

**Authors:** Harmawati Harmawati, Bunyamin Maftuh, Sapriya Sapriya, Juju Masunah, Wina Dwi Puspitasari, Jusuf Blegur

**Affiliations:** 1 Doctoral Program in Primary Education, Universitas Pendidikan Indonesia, Bandung, West Java, 40150, Indonesia; 2 Primary Teacher Education, Universitas Buana Perjuangan Karawang, Karawang, West Java, 41361, Indonesia; 3 Primary Education, Universitas Majalengka, Majalengka, West Java, 45418, Indonesia; 4 Physical Education, Health, and Recreation, Universitas Kristen Artha Wacana, Kupang, East Nusa Tenggara, 85228, Indonesia

**Keywords:** multicultural competence, online learning, teacher certification, gender, inclusive education

## Abstract

**Background:**

Multicultural education plays a crucial role in the era of globalization; however, the influence of gender and certification status on teachers’ online multicultural teaching competency (OMTC) remain underexplored. This study aims to analyze the impact of gender and certification status on teachers’ OMTC, focusing on skills, knowledge, and overall multicultural competence.

**Method:**

A mixed method approach (explanatory sequential design) was employed, involving 106 elementary school teachers from West Java Province, Indonesia (M±SD age = 37.44±9.17; teaching experience = 10.17±9.18), including 41 males (38.68%) and 65 females (61.32%), selected through a simple random sampling. Quantitative data were collected using the Multicultural Teaching Competency Scale (MTCS), and qualitative data were gathered through structured interviews. The quantitative were analyzed using an independent samples-test, while qualitative data were analyzed thematically.

**Results:**

The quantitative results indicate that gender had no significant effect on OMTC. However, certification status significantly influenced multicultural teaching skills, though it not affect multicultural teaching knowledge or the overall OMTC score. The qualitative findings identified key thematic factors contributing to these differences, including conceptual understanding, lesson planning, integration of minority cultures, empathy and reflection, and community collaboration. Certified teachers were found to be more proficient in designing inclusive learning experience and fostering empathy. However, conceptual understanding remained varied, and community collaboration was limited.

**Conclusions:**

The study concludes that certification is more effective in enhancing technical competencies, rather than conceptual or affective aspects. These finding suggest that teacher training should adopt a transformative, reflective, and contextually grounded approach to comprehensively enhance teachers’ OMTC.

## Introduction

In online learning, the implementation of multicultural education competencies still faces various challenges. These challenges include language barriers, diverse learning styles, and uneven teacher readiness to implement these approaches ( Abakirov et al., 2025). Furthermore, teachers’ lack of understanding of the concept of multicultural education prevents them from identifying events in multicultural classrooms from a cultural perspective and understanding the varying teacher perspectives on grouping students based on cultural background ( Mohiyeddini, 2024; Theeuwes et al., 2025). Some teachers have not received adequate training to integrate multicultural values into online learning materials and methods ( Wong, 2025), despite the urgency of this in a diverse country like Indonesia. Consequently, the material presented is often insensitive to students’ cultural diversity.

Another problem is that communication, limited to text or voice, along with language barriers, makes it difficult for teachers and students to understand each other’s cultural contexts. It includes the use of diction and intonation, which can have different interpretations across cultures. It can lead to misinterpretations, stereotypes, or even discrimination without students and teachers realizing it, potentially igniting horizontal conflict ( Musa, 2025). This inequality causes some students to be left behind, potentially reinforcing inequities in education. The lack of cultural representation in digital teaching materials also poses a challenge. Content is often dominated by a single cultural perspective, thus failing to represent the diversity of students, especially in a pluralistic society. Therefore, it is crucial to improve teachers’ multicultural competencies and ensure inclusivity in every aspect of online learning for every student.

Research on multicultural teaching competence (MTC) has shown significant progress in recent years, both in terms of quantity and thematic focus. First, research by Gürbüz and Yıldırım (2024) revealed that English as a Foreign Language (EFL) instructors in Turkey had very positive attitudes toward diversity, but only moderately high levels in the knowledge and skills dimensions. It indicates a gap between positive attitudes and the practical implementation of multicultural competence in the classroom. Qudsiyah et al. (2024) used a bibliometric analysis to show a sharp upward trend in publications related to MTC since 2020. This research highlights the growing attention to diversity issues in global education. Furthermore, Mensah et al. (2025) studied prospective teachers in Ghana. They found that their MTC was at a moderate level, with teaching self-efficacy, critical self-reflection, and religious inclusivity as key factors. Interestingly, this study showed that gender had a significant influence, particularly in the teaching of religious and moral subjects.

While these studies provide important insights, they still lack consistency in gender-related results and the development of materials, concepts, and measurement tools specifically for online multicultural teaching competencies. Therefore, current research needs to address these gaps to provide more contextualized and applicable guidance.

## Literature review

Gender issues in multicultural teaching have been extensively researched, but the results are inconsistent. Karacabey et al. (2019) showed no significant differences in attitudes toward multicultural education based on gender, mother tongue, or level of experience. It indicates that OMTC can develop equally among male and female teachers, provided they have access to appropriate training. However, in some contexts, as demonstrated in the study by Mensah et al. (2025), gender can be a factor influencing competence, particularly in subjects containing moral and religious values. It is important to view gender contextually and not solely as a fixed variable in OMTC studies. Likewise, competency certification status plays a crucial role in shaping teacher competency ( Amalia & Saraswati, 2018) due to its role in standardizing professionalism, professional development, and serving as a form of formal recognition of the teaching profession ( Santosa et al., 2022).

Each country has its own system for determining teacher certification, as well as the competency requirements for prospective teachers ( Fildzah, 2020). Research shows that teacher certification significantly impacts performance improvement. Consider Nurzaman’s (2019) study, which found that certification contributed approximately 23.3% to teacher performance. Yurosma et al. (2021) also found that certification positively impacts teacher professionalism. Finally, Putra and Bustami (2023) found that certified teachers had higher average performance than uncertified teachers, although both were still in the “moderate” category. Meanwhile, these findings confirm that certification can be an important instrument for improving teaching quality, although the implementation context and other support still influence its effectiveness.

Cultural diversity is an essential element and an anticipated norm in online learning environments. Teachers must develop competencies in cultural diversity awareness in students and use appropriate teaching strategies ( Hashmi & Jan, 2025) to create a positive and effective learning environment where all students can learn and thrive, regardless of their cultural or linguistic background ( Dzerviniks et al., 2024). Therefore, exploring Online Multicultural Teaching Competency (OMTC) oriented towards gender and elementary school teacher certification status is an important study in the context of 21st-century multicultural education, enabling teachers to deliver culturally responsive teaching in online classrooms ( Kim et al., 2024). It confirms that amid the rapid digitalization of learning, online multicultural teaching competency is a crucial aspect that has not been widely studied, especially at the elementary school level.

Teachers’ multicultural education teaching competencies in online learning from a gender perspective and teacher certification status as an indicator of professionalism. While most previous studies have focused on multicultural competencies in the context of junior high school education ( Lee et al., 2023) and offline learning at universities ( Gürbüz & Yıldırım, 2024), studies based on gender and competency status are important in identifying factors such as differences in technology experience and cultural sensitivity that influence the effectiveness of online multicultural teaching. While gender does not always influence intercultural communicative competence, teachers’ competencies and ethnic identities play a significant role ( Tovar-Correal & Pedraja-Rejas, 2025). These findings support more inclusive teacher professional development and the design of policies and training that ensure all teachers are prepared to teach equitably in multicultural digital contexts.

## Method

This study adopted a mixed-methods research approach with an explanatory sequential design. In this design, Creswell and Creswell (2018) explain that the initial stage is characterized by conducting a quantitative study and analyzing the results. The findings from this stage are then used as the basis for designing a qualitative study to obtain a more in-depth explanation. To facilitate the investigation, the researchers adopted the research procedure developed by Ivankova et al. (2006).

### Quantitative data collection

Respondents were 106 elementary school teachers (M±SD = 37.44±9.17) in West Java Province, Indonesia, with teaching experience of M±SD = 10.17±9.18. There were 41 male (38.68%) and 65 female teachers (61.32%) determined using a simple random sampling technique. Respondents with a high school education or equivalent were four people (3.77%), Bachelor’s education were 92 people (86.79%), and Master’s education were 10 people (9.43%). Continuing with employee status data, there were 30 contract/honorary teachers (28.30%), 45 civil servant teachers (42.45%), and 31 government employee teachers and work agreement teachers (29.25%). Of these employees, 76 teachers (71.70%) had competency certification, while the remaining 30 teachers (28.30%) had not.

The researchers collected online multicultural teaching competency data using the Multicultural Teaching Competency Scale (MTCS). This scale, developed by Spanierman et al. (2011), consists of 16 items representing two leading indicators. The first, multicultural teaching skills, comprises 10 items with a reliability value of 0.80. These include items such as “I integrate the cultural values and lifestyles of racial and ethnic minority groups into my teaching” One negative statement (item seven), “I rarely examine the instructional materials I use in the classroom for racial and ethnic bias,” was reverse-scored. The second indicator, multicultural teaching knowledge, comprises six items with a reliability value of 0.78. These included the items “I am knowledgeable about particular teaching strategies that affirm the racial and ethnic identities of all students.” Respondents responded on a five-point Likert scale: strongly disagree–strongly agree.

### Quantitative data analysis

The results of the OMTC data collection were then analyzed descriptively and comparatively to describe and simultaneously test differences in OMTC of teachers based on gender and competency certification. The results of the Shapiro-Wilk test (data normality) and Lavene (data homogeneity) found that the significance value of the OMTC variable data was >0.05 (see Table 1), which proves that the data from each sample group is normally distributed and the data variance is homogeneous. Referring to the two prerequisite tests, the statistical test uses an independent samples t-test. If the significance value is <0.05, it is concluded that there are significant differences in online multicultural teaching competency between male and female teachers or between teachers who have competency certification and those who have not, and vice versa. The entire process of collecting and analyzing quantitative data used the assistance of Google Forms, Microsoft Excel, and SPSS version 29.

**Table 1.** Data normality and homogeneity test.

Respondent groups		Shapiro-Wilk (Sig.)	Lavene (Sig.)	Conclusion
Gender	Male (n = 41)	0.080	0.391	Normal and homogeneous data
	female (n = 65)	0.095		
Teacher competency certification	Not yet (n = 30)	0.060	0.425	Normal and homogeneous data
	Yes (n = 76)	0.088		

### Connecting quantitative and qualitative phases

Based on the results of the independent samples t-test analysis based on competency certification, significant differences were evident in the first indicator, multicultural teaching skills (one-sided p = 0.025; two-sided p = 0.050). Therefore, the researchers followed up by exploring the respondents’ more contextual and clinical meanings of these differences. To guide data collection, they used a purposive sampling technique to identify six potential respondents, considering their high response rate to the multicultural teaching skills data, both those who had passed the competency certification and those who had not, and also considering gender representation. These six respondents provided practical experience with multicultural teaching skills in online learning for elementary school students. The six respondents were P (male, 38 years old), U (female, 29 years old), D (female, 39 years old), R (female, 27 years old), NAF (male, 39 years old), and HHJ (male, 36 years old). In this phase, the researchers also used semi-structured interviews for qualitative data collection to gradually explore the respondents’ perspectives and clinical meanings regarding how the competency certification material and experiences influenced their multicultural teaching skills, or vice versa.

### Qualitative data collection

At this stage, the researcher posed a series of interview questions to respondents to explore in depth the reasons behind the quantitative findings that there were significant differences in multicultural teaching skill indicators between teachers with competency certification and those without (see Table 2). Respondents’ responses were analyzed using triangulation techniques across respondents to identify thematic patterns based on the variations in experiences, perspectives, and practices they expressed. Prior to the interviews, the researcher openly explained the purpose of the study and any potential concerns the informants might have. This was done to ensure informed, voluntary participation and full responsibility for the information provided, while ensuring the protection of respondents’ rights from any form of risk or loss.

**Table 2.** Questionnaire in qualitative data collection (Example).

No	Questions	Objective
1	What changes have you made in your school environment to ensure that students from racial and ethnic minority groups have equal opportunities for success?	Identify initiatives or environmental adaptations that support equitable access and student success.
2	How do you incorporate examples of experiences and perspectives from racial and ethnic groups during your lessons?	Explore how teachers represent diverse perspectives in the teaching and learning process.
3	In your daily life as an educator, how do you promote diversity through your behavior?	Assess teachers’ concrete actions to demonstrate inclusive attitudes and respect for diversity directly.
4	How do you build strong and supportive relationships with parents from racial and ethnic minority groups?	Explore teachers’ efforts to build communication and support with diverse families.
5	How do you understand and apply culturally responsive pedagogy in your teaching practice?	Measure teachers’ awareness and ability to implement culturally sensitive teaching strategies.

### Qualitative data analysis

Qualitative data were analyzed thematically, referring to the stages recommended by Lester et al. (2020). First, researchers prepare and organize the data for analysis, where they collect, organize, and label data from interviews, observations, or documents so they are ready for analysis. Second, transcribing the data, which converts audio or video data into verbatim written text. It is crucial for easier reading and analysis of all information. Third, becoming familiar with the data involves repeatedly reading the transcripts to understand the context and begin to recognize patterns or important issues emerging from the data. Fourth, memoing the data, which involves recording the researcher’s thoughts, initial impressions, and reflections while reading the data. These memos help guide the analysis and develop themes later. Fifth, coding the data, which involves labeling meaningful sections of the data. These codes serve to organize the data into smaller, more focused units. The final stage involves moving from codes to categories and categories to themes. Similar codes are combined into categories, then developed into main themes that reflect the deeper meaning of the overall data.

### Integration of the quantitative and qualitative results

Last, this design phase integrates the quantitative and qualitative results, interpreting and explaining both study findings to complement each other. This integration can reveal the formation of new themes that explain the factors causing these significant differences, which can be used as more applicable and contextual policy recommendations to support multicultural teaching skills in various teacher competency improvement training programs.

## Findings

### Quantitative study results

The results of a gender-based descriptive analysis of the 16 Multicultural Teaching Competency items indicate that, for the multicultural teaching skills indicator, male teachers generally demonstrated slightly higher average scores for multicultural teaching skills than female teachers. Furthermore, for the multicultural teaching knowledge indicator, for the knowledge dimension, female teachers had slightly higher average scores than male teachers. They demonstrated a good understanding of various forms of diversity (statement 15), but the lowest score was in knowledge of multicultural teaching theory (statement 11).

Meanwhile, descriptive analysis based on teacher competency status shows that certified teachers have higher average scores on multicultural teaching skills indicators than non-certified teachers in almost all aspects of multicultural teaching skills. It indicates that competency certification has a positive impact on teachers’ ability to implement multicultural strategies in the classroom. Furthermore, in the multicultural teaching knowledge indicator, the difference in scores between certified and non-certified teachers in the knowledge aspect is not too significant. However, certified teachers continue to show consistent superiority, especially in understanding forms of cultural diversity. The highest score for both groups appears on statement 15, while the lowest is on statement 11, indicating the need for strengthening the theoretical aspect (see Table 3).

**Table 3.** Descriptive analysis of online multicultural teaching competency of teachers.

No	Statement	Gender (M ± SD)		Teacher competency certification (M ± SD)	
		Male	Female	Not yet	Yes
A	Multicultural teaching skill				
	1. I integrate the cultural values …	4.02 ± 0.88	3.97 ± 0.90	3.90 ± 0.71	4.03 ± 0.95
	2. I plan many activities to …	4.24 ± 0.58	4.17 ± 0.72	4.00 ± 0.59	4.28 ± 0.69
	3. I plan school events to increase …	4.39 ± 0.59	4.25 ± 0.71	4.17 ± 0.66	4.36 ± 0.67
	4. My curricula integrate topics …	4.00 ± 0.81	3.91 ± 0.90	3.57 ± 0.90	4.09 ± 0.88
	5. I make changes within the …	4.51 ± 0.60	4.23 ± 0.81	4.10 ± 0.80	4.43 ± 0.70
	6. I consult regularly with other …	4.39 ± 0.63	4.09 ± 0.76	4.03 ± 0.67	4.28 ± 0.74
	7. I rarely examine the ^a^…	3.88 ± 0.90	3.94 ± 0.79	3.97 ± 0.61	3.89 ± 0.90
	8. I often include examples of the …	4.22 ± 0.61	4.09 ± 0.72	4.07 ± 0.69	4.17 ± 0.68
	9. I often promote diversity by …	4.12 ± 0.84	4.12 ± 0.63	4.00 ± 0.64	4.17 ± 0.74
	10. I establish strong, supportive …	4.24 ± 0.66	4.14 ± 0.68	4.07 ± 0.52	4.22 ± 0.72
B	Multicultural teaching knowledge				
	11. I am knowledgeable about …	3.76 ± 0.83	3.86 ± 0.73	3.87 ± 0.51	3.80 ± 0.95
	12. I have a clear understanding of …	4.02 ± 0.57	3.97 ± 0.64	3.93 ± 0.58	4.01 ± 0.62
	13. I am knowledgeable about …	3.83 ± 0.74	4.00 ± 0.61	3.90 ± 0.55	3.95 ± 0.71
	14. I am knowledgeable of how …	3.88 ± 0.68	4.00 ± 0.53	3.90 ± 0.61	3.97 ± 0.59
	15. I understand the various …	4.07 ± 0.61	4.06 ± 0.68	4.03 ± 0.49	4.08 ± 0.71
	16. I am knowledgeable about the …	3.90 ± 0.80	3.85 ± 0.71	3.87 ± 0.57	3.87 ± 0.81

^a^ Item is reverse scored.

Continuing with the independent samples t-test analysis (gender-based). For the multicultural teaching skill indicator, the p-values of 0.126 (one-sided) and 0.252 (two-sided) indicate that the difference in skills between genders is not significant. Similarly, for the multicultural teaching knowledge indicator, the p-values obtained (0.319 and 0.638) were also far above the 0.05 significance limit, indicating that there were no differences in multicultural knowledge based on gender. For the total competency score, the p-values of 0.276 (one-sided) and 0.553 (two-sided) again indicated no significant differences (see Table 4). Overall, these results indicate that gender is not a factor that influences teachers’ multicultural teaching competency, either in terms of skills, knowledge, or overall competency.

**Table 4.** Independent samples t-Test based on gender.

Equal variances assumed	t	df	One-sided p	Two-sided p	Mean difference
Multicultural teaching skill	1.152	104	0.126	0.252	1.11670
Multicultural teaching knowledge	-0.472	104	0.319	0.638	-0.27505
Multicultural teaching competency	0.596	104	0.276	0.553	0.84165

The following analysis results of multicultural teaching competency based on teacher competency certification. First, the analysis results show that in the multicultural teaching skill indicator, the p-value is 0.025 (one-sided) and 0.050 (two-sided), which is at or below the 0.05 significance limit. It indicates a significant difference between uncertified and certified teachers, where certified teachers have higher multicultural teaching skills. The second analysis results, in the multicultural teaching knowledge indicator, the p-values (0.385 and 0.771) are well above 0.05, indicating no significant difference in multicultural knowledge between teacher groups. Third analysis, for the total score of multicultural teaching competency, the p-value (0.071 and 0.142) is also not significant, although there is a tendency for a difference (see Table 5).

**Table 5.** Independent samples t-Test based on teacher competency certification.

Equal variances assumed	t	df	One-sided p	Two-sided p	Mean difference
Multicultural teaching skill	-1.984	104	0.025	0.050	-2.05439
Multicultural teaching knowledge	-0.292	104	0.385	0.771	0.63002
Multicultural teaching competency	-1.478	104	0.071	0.142	1.51452

### Qualitative study results

Certified teachers possess in-depth multicultural teaching skills, enabling them to plan inclusive and culturally responsive learning. They actively integrate minority cultures into core teaching materials, demonstrate empathy, and critically reflect on teaching practices. Furthermore, certified teachers build collaborations with local communities, utilize resources to support diverse learning, and create equitable and supportive learning environments for all students.

***Depth of conceptual understanding of multiculturalism***

Teachers with competency certification can systematically integrate the values of inclusivity, cultural representation, and diverse perspectives into their learning. They recognize that multicultural education is not simply about introducing various cultures but also about creating an equitable, safe, and supportive learning environment for all students, regardless of background. They implement culturally responsive pedagogy by adapting teaching approaches based on students’ identities and experiences. These teachers are able to recognize differences in learning styles, communication patterns, and values held by minority students, and adapt strategies to ensure all students feel valued and empowered. The use of representative teaching materials and the facilitation of discussions that support tolerance are their characteristics, as stated in the following reference code.

I implement culturally responsive pedagogy by adapting my teaching methods to students’ backgrounds, experiences, and cultural identities, so they feel valued, connected, and motivated. I strive to understand the values, traditions, languages, and learning habits of students from diverse groups. For example, I learned that some students are accustomed to learning orally through stories at home, so I incorporated storytelling into my classroom. (P/male/38 years old).

To integrate the cultural values and lifestyles of minority groups into my teaching, I use subject matter relevant to students’ backgrounds, create an inclusive learning environment, and engage families and communities. For example, I teach local history and traditions through stories or festivals, and I use various media such as music, dance, and art to introduce minority cultures to all students. (NAF/male/39 years old).

Teacher competency certification has been shown to significantly contribute to broadening teachers’ theoretical insights into students’ multicultural education practices. Teachers who have undergone the certification process have a more structured understanding and awareness of the importance of managing diversity in education. Through training, material provision, and standardized evaluation, certification helps build a strong conceptual and practical foundation regarding the values of inclusivity, equity, and social justice. This understanding encompasses cultural representation in teaching materials, cross-cultural perspectives, and the application of culturally responsive pedagogy. Certified teachers not only recognize racial, ethnic, and cultural differences but are also able to respond reflectively and systematically through lesson planning, strategy selection, and inclusive social interactions. With certification, teachers are better equipped to become facilitators who are aware of multicultural values, creating welcoming learning spaces and supporting critical awareness of diversity and educational justice.

***Planned multicultural teaching strategies***

Certified teachers more frequently use planned, contextualized learning strategies tailored to students’ cultural backgrounds. They do not rely solely on general methods but also design learning that is relevant to the lived experiences of students, particularly those from minority groups. Learning planning is carried out carefully, taking into account multicultural values, such as inclusion, empathy, and diverse perspectives. Strategies typically involve cross-cultural discussions, projects based on local wisdom, and the use of media representing various ethnicities and cultures. This approach helps students feel emotionally and cognitively connected to the material being taught.

I integrate the cultural values and lifestyles of racial and ethnic minority groups into my teaching through an inclusive learning approach. For example, to ensure that daily lessons do not focus solely on the majority group, educators can incorporate learning about the history of each region, explore local folklore, or create poems or songs relevant to the students’ regions of origin. (U/female/29 years old).

To integrate the cultural values and lifestyles of minority groups into teaching, educators can use subject matter relevant to students’ backgrounds, create an inclusive learning environment, and engage families and communities. Examples include teaching local history and traditions through stories or festivals, and using various media such as music, dance, and art to introduce minority cultures to all students. (NAF/male/39 years old).

Competency certification strengthens teachers’ ability to design multicultural learning in a structured and systematic manner. Teachers no longer respond to diversity spontaneously or situationally, but can develop consistent and sustainable learning strategies. With the theoretical understanding gained through the certification process, teachers can integrate multicultural values into learning objectives, materials, methods, and evaluations. It enables the creation of an inclusive learning environment that is relevant to students’ cultural contexts and fosters a deeper and more sustainable appreciation of differences.

***Integration of minority values and perspectives in the curriculum***

Certified teachers more frequently use alternative and contextual teaching materials rich in cultural values. These include folktales from various regions, the history of minority groups, and true stories relevant to students’ lives. The use of these materials not only enriches learning but also helps students understand and appreciate cultural diversity more deeply. In this way, teachers can create authentic and meaningful learning experiences while fostering tolerance and empathy among students from different backgrounds. This approach demonstrates certified teachers’ awareness of implementing multicultural education effectively.

Suppose there are children from a minority group who create stories about their hometowns. In that case, we as educators allow them to convey every explanation in their assignments so that it reaches the majority of their classmates. After that, we provide additional reminders about the cultural diversity around us that we should know and appreciate. (U/female/29 years old).

I incorporate folktales, songs, and literary works from various ethnicities/minorities into my teaching materials. For example, when teaching Indonesian, in addition to the story of Malin Kundang, I also introduce folktales from the *Baduy* or *Dayak* tribes, then encourage students to compare their moral values. (P/male/38 years old).

Certified teachers demonstrated greater preparedness in comprehensively integrating minority group perspectives into core teaching materials. They did not simply include minority cultural elements as supplementary or supplementary activities, but instead made them an integral part of the learning process. This approach ensured that cultural diversity became a consistent and integrated theme in the curriculum, enabling students to gain a deeper and more holistic understanding of cultural differences. Thus, certified teachers were able to create an inclusive learning environment and appreciate diversity tangibly and sustainably.

***Empathy, tolerance, and reflective attitude***

Both groups of teachers demonstrated empathy and goodwill in addressing diversity in the school environment. However, certified teachers tended to demonstrate a higher level of reflection and stronger contextual awareness. They were able to link the values of inclusivity to daily learning practices in a more purposeful manner. Certified teachers actively reviewed, evaluated, and developed their teaching approaches to become more inclusive and responsive to the needs of students from diverse backgrounds. It was reflected in the selection of diverse learning materials, participatory teaching methods, and activities that fostered empathy and appreciation for cultural differences.

I build strong and supportive relationships with parents from racial and ethnic minority groups by respecting their customs and not allowing differences to become barriers to good relationships, for example, by recognizing and respecting their culture and involving parents in the community in every school activity. (Uni/female/29 years old).

The historical experiences of racial and ethnic minority groups can significantly impact students’ learning, as they connect lessons to real-life situations. Minority history can serve as a mirror for understanding current social issues. For example, discussing the history of Chinese Indonesians can help students understand the importance of tolerance in today’s economic and cultural diversity. (P/male/38 years old).

Certification competencies appear to encourage teachers to critically and reflectively review their teaching practices, rather than simply performing routine “good deeds.” Certified teachers are better able to evaluate the effectiveness of their multicultural learning strategies, understand their students’ contexts in depth, and connect diversity values to clear learning objectives. Thus, competency certification not only reinforces good intentions but also enhances professional awareness and the quality of teaching in a more systematic and meaningful multicultural context.

***Leveraging community and surrounding resources***

In online classes, certified teachers actively leverage community resources by building strong relationships with community leaders and involving various local institutions in the learning process. They understand the importance of collaborating with the surrounding community to enrich teaching materials and provide authentic and contextual learning experiences for students. This approach not only strengthens students’ understanding of cultural diversity in online classes but also builds social networks that support inclusivity and respect for minority groups more tangibly and sustainably.

I utilize these resources to support student learning. For example, Chromebooks, infocus cameras, speakers, and infocus screens. I use them as a means for students to learn about different cultures by watching films or videos and singing along to regional songs. (U/female/29 years old).

These community resources include institutions, figures, places, and community activities that can support learning. For example, village/sub-district halls and community leaders can be sources of local wisdom values. Art studios or cultural groups can be places to learn dance, traditional music, or crafts. Religious institutions (mosques, churches, temples, monasteries) can be sources of moral values, tolerance, and harmony. Environmental communities such as farmer groups, waste banks, or nature lovers can be sources of science learning. (P/male/38 years old).

Therefore, it was concluded that certified teachers tend to have broader social networks and engagement with various communities, community leaders, and local institutions. This engagement strengthens their ability to teach effectively and contextually with a multicultural approach in online classes. Through these relationships, teachers can access diverse cultural resources, provide students with authentic experiences, and enrich teaching materials with local perspectives. Thus, these strong social networks are a crucial supporting factor in developing deeper and more integrated multicultural teaching skills.

### Integration of the quantitative and qualitative results

The results of the study indicate that teacher competency certification significantly impacts technical skills in online multicultural teaching, but has no significant impact on conceptual knowledge or total competency scores. This finding is supported by qualitative data, which indicates that certified teachers tend to be able to design inclusive learning, integrate minority cultures, and demonstrate empathy and reflection. However, their conceptual understanding remains diverse, and collaboration with the community is uneven. Therefore, teacher professional development needs to focus on strengthening the substance of multiculturalism through a reflective and contextual approach, rather than being based on demographic characteristics.

Table 6 shows significant differences in OMTC between certified and uncertified teachers. Uncertified teachers tended to possess a more general, pragmatic, and technical understanding, with lesson planning that was ofter situational and unstructured. The integration of minority cultures was partial and not yet systemic, while empathy and reflection were more intuitive, lacking formal self-evaluation. Collaboration with the community was also limited. In contrast, certified teachers demonstrated a deeper understanding grounded in multicultural pedagogical theory. Their lesson planning was more systematic and contextually relevant, and their integration of minority cultures was more comprehensive. Additionally, certified teachers exhibited greater self-evaluation, more conscious empathy, and were more active in utilizing social networks and collaborating with the community.

**Table 6.** Thematic factors of online multicultural teaching competency between uncertified and certified teachers.

Thematic factors	Teachers do not yet have competency certification	Teachers have a competency certification
Conceptual understanding	General, more pragmatic and technical	Strong, based on multicultural pedagogical theory
Learning planning	Situational and unplanned	Systematic and contextual
Integration of minority cultures	Partial and not yet systematic	Comprehensive in teaching materials
Empathy and reflection	Intuitive, lacks self-evaluation	Aware, contextual, and reflective
Collaboration with the community	Minimal external involvement	Actively utilize social networks and communities

## Discussion

This study shows no statistically significant differences between male and female teachers in online multicultural teaching competency, either in terms of skills, knowledge, or total competency scores. This finding indicates that gender is not a determining factor in mastering multicultural teaching competency in the context of online learning in Indonesia. This finding aligns with the study by O’Donnell et al. (2025), which also found no gender differences in OMTC scores among online anti-bias training participants, even though the majority of participants were female. However, this finding differs from the study by Mensah et al. (2025), which identified a significant gender influence in the teaching of religious and moral subjects, suggesting that the influence of gender on multicultural teaching competency can vary based on social and cultural contexts. Therefore, this study strengthens the argument that gender is not a primary variable in determining OMTC, particularly in the context of online education in Southeast Asia.

Furthermore, the results also indicate that teacher competency certification significantly influences the skills aspect of online multicultural teaching, but does not significantly influence the knowledge aspect or the total competency score. These findings indicate that the current certification program places greater emphasis on developing practical skills in the context of online teaching. At the same time, the conceptual and reflective aspects related to OMTC remain suboptimal. It aligns with the findings of Baldan Babayiğit et al. (2025), who emphasized the importance of practice-based and collaborative teacher training, but underemphasized in-depth conceptual understanding. Furthermore, Cancino and Nuñez (2023) added that even though teachers possess high levels of multicultural awareness and empathy, they still face difficulties in implementing effective intercultural communication online. Therefore, this study recommends that certification materials be expanded by incorporating conceptual and reflective aspects to strengthen teacher competency in online multicultural learning.

The qualitative findings of this study identified five key themes that differentiate certified teachers in online multicultural teaching competency: (1) conceptual understanding, (2) inclusive online lesson planning, (3) integration of minority cultures into online materials, (4) empathy and reflection toward student diversity, and (5) collaboration with the virtual community. These themes illustrate certified teachers’ abilities to manage cultural diversity in online learning environments effectively. These findings are consistent with Markey et al. (2023), who emphasized the need for sensitive and proactive culturally responsive pedagogy in diverse online learning environments. Furthermore, Kumi-Yeboah et al. (2020) recommended enhancing the capacity of online instructors to integrate multicultural content, which can support the academic success of students from diverse cultural backgrounds. These findings emphasize that empathy, reflection, and online community collaboration are essential components in developing OMTC.

Furthermore, Chen (2024) demonstrated that field-based learning experiences connected to real-life social issues can enhance students’ critical awareness and multicultural action, which is relevant for the practical development of teachers’ OMTC. Sadykova (2014) also highlighted the importance of social support and peer relationships as mediators of cultural knowledge, particularly in the context of cross-cultural online learning. These studies reinforce the finding that OMTC development must include socio-emotional aspects and in-depth community collaboration. Globally, bibliometric analysis by Qudsiyah et al. (2024) and a thematic review Iskandar et al. (2025) indicate a trend of increasing attention to online multicultural teaching, but identify a lack of conceptual consistency and adequate measurement tools for online contexts. This study fills this gap by presenting a thematic framework based on empirical data and local contexts, which can serve as a foundation for future OMTC research and practice.

Overall, these findings provide an important contribution to the OMTC literature by combining global and local perspectives. This research also emphasizes the importance of evaluating and developing teacher certification programs to focus not only on practical skills but also on the conceptual, reflective, and collaborative aspects essential for online multicultural learning. This effort is crucial given the unique challenges and dynamics of cultural diversity in today’s online and distributed learning. Globalization and advances in communication technology have increased diversity and complexity, including in the world of education. Diversity is now present in the classroom. This situation exacerbates the need for multicultural competencies ( Abakirov et al., 2025; Patras et al., 2025), which are crucial for fostering an inclusive and responsive learning environment for multiculturalism ( Qudsiyah et al., 2024; Thresia et al., 2025). Multicultural educational competencies are crucial in online learning because the digital environment brings together students from diverse cultural, religious, ethnic, and linguistic backgrounds.

## Conclusion and implications

The study results showed gender is not the primary determinant of OMTC mastery. Instead, teacher certification status is a significant differentiator, particularly in improving multicultural teaching skills. However, certification has not shown a significant impact on multicultural teaching knowledge, which encompasses an understanding of the basic principles of multiculturalism. Qualitative data also revealed that teachers’ conceptual understanding remains diverse. Some teachers have demonstrated the ability to design inclusive learning, integrate minority cultures, and foster empathy and reflection, but these practices are not widespread. Collaboration with the community is also minimal. Thus, certification has been effective in improving technical skills, but not optimal in fostering conceptual understanding and reflective attitudes.

Several important implications of the research findings for policy development and programs to improve OMTC. First, a review of the certification curriculum is needed to ensure a balance between strengthening practical skills and mastery of multicultural concepts and values. Reflective and theoretical materials should be an integral part of the training and assessment process. Second, post-certification training programs and continuing professional development should be designed to deepen teachers’ understanding of cultural diversity and equip them with empathy, critical thinking, and reflection in their teaching practice. Third, because no significant differences were found between male and female teachers, training can be focused on a competency-based approach, rather than on gender or other demographic characteristics. Finally, certification bodies need to evaluate online multicultural competency assessment indicators to ensure they reflect the full dimensions of knowledge, skills, and attitudes.

## Ethics and consent

This study received approval from the Research Ethics Committee of Universitas Buana Perjuangan Karawang (Approval Letter No. 750/LPPM/IV/2025, dated April 8, 2025). Informed written and verbal consent was obtained from all participants voluntarily. To ensure the protection of participants’ rights and privacy, all collected data will be kept confidential and used solely for research purposes.

## Data Availability

Open Science Framework (OSF): Quantitative data on teacher competencies in online multicultural teaching. https://doi.org/10.17605/OSF.IO/2DXCY ( Harmawati, Maftuh, Sapriya, Masunah, et al., 2026b) The project contains the following underlying data: Data.xlsx. Anonymous responses from 106 respondents regarding online multicultural teaching competencies, using a five-point Likert scale, were coded as follows: “Strongly disagree” was coded as 1, and “Strongly agree” was coded as 5. Data are available under the terms of the Creative Commons Zero “No rights reserved” data waiver (CC0 1.0 Universal). Open Science Framework (OSF): Qualitative data (sample) on teacher competency in online multicultural teaching. https://doi.org/10.17605/OSF.IO/EC8XZ ( Harmawati, Maftuh, Sapriya, Masunah, et al., 2026a) The project contains the following underlying data: Data.xlsx. Anonymous responses from 6 respondents regarding online multicultural teaching competencies. Data are available under the terms of the Creative Commons Zero “No rights reserved” data waiver (CC0 1.0 Universal). Open Science Framework (OSF): Multicultural Teaching Competency Scale (MTCS). https://doi.org/10.17605/OSF.IO/G3H8X ( Harmawati, Maftuh, Sapriya, Msaunah, et al., 2026) This project contains the following extended data. Questionnaire. MCTS = Multicultural Teaching Competency Scale; F1 = multicultural teaching skill, item-10 (M = 4.36, SD = 0.73, α = 0.80, variance accounted for = 32.89%); F2 = multicultural teaching knowledge, item 11-16 (M = 4.80, SD = 1.01, α = 0.78, variance accounted for = 8.76%). Means and standard deviations of the scales are divided by the number of items in each. Data are available under the terms of the Creative Commons Zero “No rights reserved” data waiver (CC0 1.0 Universal).
