# Supplementary material for: Revealing gendered patterns and teacher competency status in online multicultural teaching competency
Source: F1000Res. 2026 May 26;15:238. Originally published 2026 Feb 11. [Version 2] doi: 10.12688/f1000research.177259.2 (PMC13044540; doi:10.12688/f1000research.177259.2)
Supplement: Peer review history

# Reviewer report: Reviewer response for version 2

**DOI:** 10.5256/f1000research.201468.r488531

**Published:** 2026-06-08

**Authors:** Maksal Minaz

**Affiliations:** 1 Abdul Wali Khan University Mardan, Mardan, Pakistan

The article has now been accepted after the revisions made by the authors.

Is the work clearly and accurately presented and does it cite the current literature?

Yes

If applicable, is the statistical analysis and its interpretation appropriate?

Partly

Are all the source data underlying the results available to ensure full reproducibility?

Yes

Is the study design appropriate and is the work technically sound?

Yes

Are the conclusions drawn adequately supported by the results?

Yes

Are sufficient details of methods and analysis provided to allow replication by others?

Yes

Reviewer Expertise:

Educational Research, Blended learning, teaching strategies, qualitative and quantitative research and Educational psychology

I confirm that I have read this submission and believe that I have an appropriate level of expertise to confirm that it is of an acceptable scientific standard.

# Reviewer report: Reviewer response for version 2

**DOI:** 10.5256/f1000research.201468.r488529

**Published:** 2026-06-03

**Authors:** Nansia Kyriakou

**Affiliations:** 1 Frederick University, Nicosia, Nicosia, Cyprus

The manuscript has improved considerably following revision, particularly in terms of conceptual clarity, theoretical framing, and the interpretation of findings. The topic remains timely and relevant, and the Indonesian context offers a valuable contribution to the literature on multicultural and online teaching competencies. The qualitative findings are meaningful and help illuminate the multidimensional nature of OMTC.

 Τhe manuscript is now significantly stronger and has the potential to make a useful contribution to the field

Is the work clearly and accurately presented and does it cite the current literature?

Partly

If applicable, is the statistical analysis and its interpretation appropriate?

Partly

Are all the source data underlying the results available to ensure full reproducibility?

Partly

Is the study design appropriate and is the work technically sound?

Partly

Are the conclusions drawn adequately supported by the results?

Partly

Are sufficient details of methods and analysis provided to allow replication by others?

Partly

Reviewer Expertise:

Bilingualis, Multilingualis, Intercultural Studies

I confirm that I have read this submission and believe that I have an appropriate level of expertise to confirm that it is of an acceptable scientific standard.

# Reviewer report: Reviewer response for version 1

**DOI:** 10.5256/f1000research.195453.r468039

**Published:** 2026-04-01

**Authors:** Maksal Minaz

**Affiliations:** 1 Abdul Wali Khan University Mardan, Mardan, Pakistan

**1. Is the work clearly and accurately presented, and does it cite the current literature?**

 To make the study **more concise, theoretically grounded, and visually appealing**, the researchers need two things:

1. A **strong theoretical framework** (what theories support the study)
2. A **clear conceptual framework** (how the variables are connected)

Since your study (from earlier context) relates to **leadership strategies, conflict resolution, and institutional progress**, here is a **polished, thesis-ready version** you can adapt:

**4. If applicable, is the statistical analysis and its interpretation appropriate?**

 The independent samples t-test results are adequately reported with p-values; however, the inclusion of effect size (e.g., Cohen’s d) is recommended to evaluate the practical significance of the findings.

 While the manuscript claims to address a research gap, it does not clearly demonstrate how it differs from existing studies. The authors should strengthen the justification of their contribution by explicitly identifying what is new, unique, and significant in the present research.

 While both quantitative and qualitative data are presented, their integration remains insufficient. The use of joint displays is recommended to align and compare findings effectively. Moreover, clearer meta-inferences should be drawn to explain how the combined results provide deeper insights into the research problem.

 The study claims the use of simple random sampling; however, the absence of a defined sampling frame raises concerns about transparency. The researchers should explicitly clarify the target population and the procedure used to select respondents.

**Language Issues:**

 The manuscript contains several grammatical and syntactical errors that affect readability. For example, phrases such as *“it not affect”* should be revised to *“it does not affect,”* and *“These finding suggest”* should be corrected to *“These findings suggest.”* A thorough language review is recommended to ensure clarity and academic tone.

**Structure:**

 The organization of the manuscript can be improved. Some paragraphs are excessively long, which reduces readability. Additionally, there is noticeable repetition in the qualitative findings section, which should be minimized to enhance conciseness and coherence.

**Tables:**

 The tables require improvement in formatting and presentation. Some tables lack clarity, making it difficult for readers to interpret the data effectively. It is recommended to standardize table formatting and provide clearer labels, titles, and notes where necessary.

Is the work clearly and accurately presented and does it cite the current literature?

Yes

If applicable, is the statistical analysis and its interpretation appropriate?

Partly

Are all the source data underlying the results available to ensure full reproducibility?

Yes

Is the study design appropriate and is the work technically sound?

Yes

Are the conclusions drawn adequately supported by the results?

Yes

Are sufficient details of methods and analysis provided to allow replication by others?

Yes

Reviewer Expertise:

Educational Research, Blended learning, teaching strategies, qualitative and quantitative research and Educational psychology

I confirm that I have read this submission and believe that I have an appropriate level of expertise to confirm that it is of an acceptable scientific standard, however I have significant reservations, as outlined above.

# Reviewer report: Reviewer response for version 1

**DOI:** 10.5256/f1000research.195453.r462825

**Published:** 2026-03-25

**Authors:** Nansia Kyriakou

**Affiliations:** 1 Frederick University, Nicosia, Nicosia, Cyprus

**Summary of the article**

 This manuscript examines online multicultural teaching competency (OMTC) among elementary school teachers in West Java, Indonesia, focusing on gender and certification status. Using an explanatory sequential mixed-methods design, the authors collected quantitative data from 106 teachers with the MTCS and followed this with interviews involving six participants. The topic is relevant and timely, and the mixed-methods design is potentially appropriate. The paper also has practical value for teacher development and certification policy. However, important conceptual, methodological, and reporting issues need to be addressed.

*1. Is the work clearly and accurately presented and does it cite the current literature?*

**Partly**

 The topic is clearly important, and the manuscript cites recent literature. However, the main construct, OMTC, is not clearly defined. It is introduced as a theme rather than as a precise concept. The paper should also differentiate OMTC from broader multicultural teaching competence and explain more clearly how and whether online teaching changes the construct. In addition, the literature review would benefit from a stronger theoretical framework, and the manuscript should explicitly state its research question or questions.

*2. Is the study design appropriate and is the work technically sound?*

**Partly**

 The mixed-methods design is suitable in principle. However, the main concern is measurement: the study claims to assess online multicultural teaching competency, but it uses the MTCS without clearly explaining whether the scale was adapted for online teaching. This creates uncertainty about whether OMTC was actually measured. There is also some inconsistency in the qualitative description, as the paper refers to both structured and semi-structured interviews.

*3. Are sufficient details of methods and analysis provided to allow replication by others?*

**Partly**

 Some useful details are provided, but not enough for full replication. The manuscript should explain more clearly how the sampling was carried out, whether the instrument was translated or adapted, and how reliability performed in the current sample. The interview protocol is not sufficiently reported, and the integration of quantitative and qualitative phases needs clearer explanation (how they complement each other). Demographic information would also be easier to follow in a table.

*4. If applicable, is the statistical analysis and its interpretation appropriate?*

**Partly**

 The use of group comparison tests is generally reasonable, but the reporting and interpretation need improvement. The paper reports one-sided and two-sided p-values without sufficient justification. In places, the interpretation of the qualitative data goes beyond the results and would be better placed in the discussion. The findings section should remain more descriptive, with interpretation reserved for later.

*5. Are all the source data underlying the results available to ensure full reproducibility?*

**Partly**

 It is not clear from the manuscript whether the underlying quantitative dataset, coding outputs, or qualitative materials are available. If data cannot be shared fully for ethical reasons, the authors should provide a clear data availability statement explaining what is available and under what conditions.

*6. Are the conclusions drawn adequately supported by the results?*

**Partly**

 The conclusions are partly supported by the results, especially regarding the limited role of gender and the association between certification and the skills dimension. However, some claims are stronger than the evidence allows. In particular, the manuscript should be more cautious about suggesting that certification improves OMTC when the design is not causal and when the measure’s online specificity is unclear.

***Additional comments***

 The paper has several strengths: the topic is relevant, the Indonesian context is valuable, and the qualitative themes are potentially useful. To strengthen the manuscript, I would also suggest presenting participant demographics in a table, organizing findings around the research questions, and explaining more clearly how the qualitative interviews complement the quantitative results. Overall, the study has promise, but the issues above should be addressed to make the article scientifically sound.

Is the work clearly and accurately presented and does it cite the current literature?

Partly

If applicable, is the statistical analysis and its interpretation appropriate?

Partly

Are all the source data underlying the results available to ensure full reproducibility?

Partly

Is the study design appropriate and is the work technically sound?

Partly

Are the conclusions drawn adequately supported by the results?

Partly

Are sufficient details of methods and analysis provided to allow replication by others?

Partly

Reviewer Expertise:

Bilingualis, Multilingualis, Intercultural Studies

I confirm that I have read this submission and believe that I have an appropriate level of expertise to confirm that it is of an acceptable scientific standard, however I have significant reservations, as outlined above.

# Reviewer report: Reviewer response for version 1

**DOI:** 10.5256/f1000research.195453.r465531

**Published:** 2026-03-19

**Authors:** Zahra Sadat Mohajeri

**Affiliations:** 1 University of Salerno, Salerno, Italy

This manuscript addresses an important and timely issue in the field of online multicultural education, offering valuable contributions to the literature. The use of a mixed-methods approach with an explanatory sequential design is a notable strength, as it enables a more comprehensive understanding of the phenomenon by integrating quantitative and qualitative findings. The application of a standardized instrument (MTCS) and appropriate statistical analysis (independent samples t-test) reflects sound methodological rigor. The findings—particularly the lack of significant gender differences and the positive impact of certification on teaching skills—provide meaningful insights. Moreover, the qualitative results enrich the study by identifying key themes such as inclusive lesson planning, empathy, and community collaboration, which deepen the interpretation of the quantitative outcomes.

 However, several aspects of the manuscript require further refinement. The integration between quantitative and qualitative findings could be more explicitly articulated to strengthen the overall coherence of the study. Additionally, the sample is limited to one province in Indonesia, which constrains the generalizability of the results and should be more clearly acknowledged as a limitation. The finding that certification does not significantly influence theoretical knowledge warrants a more critical and in-depth discussion in relation to existing literature. Finally, the study would benefit from a stronger and more clearly defined theoretical framework for “online multicultural teaching competency” to enhance conceptual clarity and strengthen the academic contribution.

Is the work clearly and accurately presented and does it cite the current literature?

Yes

If applicable, is the statistical analysis and its interpretation appropriate?

Yes

Are all the source data underlying the results available to ensure full reproducibility?

Yes

Is the study design appropriate and is the work technically sound?

Yes

Are the conclusions drawn adequately supported by the results?

Yes

Are sufficient details of methods and analysis provided to allow replication by others?

Yes

Reviewer Expertise:

education.inclusion. multiculturalism

I confirm that I have read this submission and believe that I have an appropriate level of expertise to confirm that it is of an acceptable scientific standard.
